# Bridging Theory and Practice in Emergency Nursing: A Discussion Paper on Peripheral intravenous catheter Management Using Tanner's and Chinn & Kramer's Models

**DOI:** 10.1177/23779608261433137

**Published:** 2026-04-22

**Authors:** Joana Moreira Ferreira Teixeira, Gillian Ray-Barruel, Gisela Mosca Teixeira, Candida Durao, Celeste Bastos, Maria do Rosário Pinto

**Affiliations:** 1 School of Medicine and Biomedical Sciences (ICBAS), 26706 Porto University (UP), Porto, Portugal; 2 RISE-Health, Escola Superior de Enfermagem, Universidade do Porto, Porto, Portugal; 3 Univ Coimbra, UICISA E, ESEUC, Coimbra, Portugal; 4 Nursing Research Innovation and Development Centre of Lisbon (CIDNUR), School of Nursing, Universidade de Lisboa. Lisbon. Portugal; 5 Herston Infectious Diseases Institute, 1974 Metro North Health, Brisbane, Queensland, Australia; 6 School of Nursing, Midwifery and Social Work, The University of Queensland, Brisbane, Queensland, Australia; 7 Alliance for Vascular Access Teaching and Research (AVATAR), 5723 Griffith University, Brisbane, Queensland, Australia; 8 Diaverum, Linda-a-Velha, Portugal; 9 School of Nursing, Universidade de Lisboa, Lisbon, Portugal

**Keywords:** Clinical decision-making, emergency department, evidence-based practice, nursing theory, peripheral venous catheterization

## Abstract

**Introduction:**

Nursing management of peripheral intravenous catheters remains suboptimal and often lacks integration with underpinning theory. Therefore, it is essential to reflect on the persistent gap between theory and practice to enhance patients’ outcomes in this area.

**Objectives:**

To conceptually evaluate the integration of Tanner's Clinical Judgment Model using Chinn and Kramer's criteria, and to explore the integration of these two complementary frameworks with Kramer's Ways of Knowing for peripheral intravenous catheter decision-making and patient outcomes improvement in the emergency department.

**Methods:**

A discussion paper, using Tanner's Clinical Judgment and Chinn & Kramer's Models, to bridge theory and practice, in peripheral intravenous catheter management in the emergency departments, done in October 2024, through consensus among authors, in interactive rounds.

**Results:**

According to Chinn and Kramer's model, which advocates evidence-informed and theory-based practice, the absence of theory in nursing education and practice is detrimental to the discipline. Tanner's model supports the integration of evidence into practice, and Chinn and Kramer's framework guides nurses in refining their decisions. In deciding whether to insert a peripheral intravenous catheter, emergency department nurses engage in cognitive and reflective processes, consult evidence-based guidelines, and integrate empirical evidence with personal and ethical considerations to ensure technically sound, empathetic, and ethically grounded patient care.

**Conclusions:**

This article analyses and promotes critical reflection on advanced practice nursing decision-making in the emergency department, exemplified by the holistic, evidence-informed processes that nurses use in peripheral intravenous catheter management. Future research is needed to evaluate the effectiveness of these models in education and clinical reasoning in specific emergent situations.

## Introduction

Peripheral intravenous catheters (PIVC) are the most used vascular devices in acute care, with an estimated 70% of adult emergency department (ED) patients receiving at least one within their first 24 hours of treatment (Alexandrou et al., 2018; Marsh et al., 2024). Although PIVC insertion is often deemed routine, premature device failure, defined as any unplanned removal before completing therapy due to infiltration, occlusion, dislodgement, phlebitis, or suspected infection, occurs in 32% to 44% of ED-placed PIVCs within 72 hours (Marsh et al., 2021; Zingg et al., 2023). Each failure disrupts treatment, adds extra costs, and risks patients experiencing unnecessary pain and infection (Høvik et al., 2019; Palese et al., 2016; Zingg et al., 2023). Since nurses are mainly responsible for managing this process, including catheter insertion dwell-time monitoring, and timely removal, PIVC performance is now recognized as a nursing-sensitive quality indicator in several national safety programmes (Capdevila et al., 2016; Gunasegaran et al., 2018; Simonetti et al., 2019; Zingg et al., 2023).

Despite the publication of comprehensive, evidence-based guidelines (World Health Organization, 2024), observational audits of PIVC management reveal significant variation in key practices. Inappropriate site selection, oversized catheters, non-transparent dressings, prolonged dwell times, and delayed removal of idle catheters are prevalent (Alexandrou et al., 2018; Ray-Barruel & Alexander, 2023; Walker et al., 2022), showing that practices related to PIVC management often remain suboptimal (Capdevila et al., 2016; Gunasegaran et al., 2018; Kelly & Egerton-Warburton, 2014; Marsh et al., 2021, 2024; Ray-Barruel & Alexander, 2023; Simões et al., 2022; Xu et al., 2023; Zingg et al., 2023).

These findings underline a persistent phenomenon that deserves attention: An undeniable gap between theory and practice. This gap is not merely an implementation failure but represents a complex interplay between cognitive processes, contextual pressures, and the multifaceted nature of nursing knowledge. To move beyond descriptive audits of practice variation, a deeper theoretical understanding of how nurses make real-time decisions is required. Such an understanding can inform targeted interventions that address the root causes of the evidence–practice gap, rather than its symptoms.

Evidence-based best practice guidance and knowledge acquisition do not automatically translate into context-aware actions in a busy, time-constrained healthcare environment, such as the ED. Some barriers, such as time constraints in the ED, a lack of training in reflective practice, or personal or institutional barriers, could impact nurses’ clinical practices (Teixeira et al., 2025a). Understanding the gap between knowledge and practice pertaining to PIVC management deserves serious analysis. To address this gap, this paper employs two complementary theoretical frameworks to explore this evidence-knowledge-practice gap. First, the study describes Tanner's Clinical Judgment Model (CJM), a theoretical framework outlining the cognitive processes involved in decision-making (Tanner, 2006; Tanner et al., 2022). Next, Chinn and Kramer's Ways of Knowing (Chinn et al., 2022) theory extends this framework by placing each clinical decision within five epistemic domains—empirical, ethical, aesthetic, personal, and emancipatory—that justify or restrict nursing actions. Chinn and Kramer's evaluative criteria (purpose, clarity, simplicity, generality, accessibility, and significance) are then applied sequentially to assess how well Tanner's CJM aligns with PIVC-related care. The analysis further demonstrates how the CJM translates the otherwise abstract Ways of Knowing into practice within a complex ED setting. Integrating Tanner's CJM with Chinn & Kramer's model for PIVC management in the ED is underexplored in current literature.

Therefore, this discussion paper aims to provide a novel theoretical synthesis. By critically appraising Tanner's CJM through a rigorous epistemic lens and demonstrating its integration with a broader philosophy of nursing knowledge, a comprehensive framework is developed. This framework is designed to make the implicit processes of expert PIVC management explicit, thereby creating a roadmap for education, practice improvement, and future research.

To operationalize this aim, this article fulfils a dual function, combining analytical and pragmatic elements, aiming to:

(1) Critically appraise Tanner's CJM using the rigorous evaluative criteria proposed by Chinn and Kramer: Purpose, clarity, simplicity, generality, accessibility, and significance;

(2) Demonstrate how integrating Tanner's CJM with Chinn and Kramer's multidimensional Ways of Knowing can inform actionable strategies to improve decision-making for PIVC management in the ED.

The proposed framework transcends the traditional view of PIVC care as a series of isolated technical tasks, instead framing it as a domain of sophisticated clinical judgment that requires ongoing reflection, focused education, and continuous quality improvement.

## Background

### Purpose and Central Concepts of the Two Models

#### Tanner's CJM

Tanner's CJM, which encompasses the 2006 and 2022 frameworks (Tanner, 2006; Tanner et al., 2022) portrays nursing decision-making as an iterative process involving four key components: (1) *Noticing* patient signs, (2) *interpreting* their significance, (3) *responding* with context-aware interventions, and (4) *reflecting* on outcomes (Tanner, 2006). The updated CJM-2022 (Tanner et al., 2022) retains this architecture but is informed by interpretative and cognitive science studies in acute care. It now codifies intuition as experientially honed pattern recognition, foregrounds comprehensive knowing of the patient, and embeds real-time reflection-in-action and continuous surveillance for early deterioration. Additionally, the updated model incorporates advanced technologies and decision support systems to enhance nurses’ clinical judgment.

The CJM also acknowledges nursing expertise. According to Tanner et al. (2022), nurses have expectations based on past experiences and must address them through the systematic assessment of all reasoning processes. Factors that positively impact nursing surveillance include nursing experiences, academic level, and knowing the patients. The CJM's applicability also extends to nursing education, particularly in simulation and debriefing practices. It emphasizes the incorporation of evidence-based practices and shared decision-making, with a focus on interprofessional collaboration, especially in critical care (Tanner et al., 2022).

#### Chinn and Kramer's Ways of Knowing

The Chinn and Kramer Ways of Knowing (Chinn and Kramer, 2023; Chinn et al., 2022) is a robust framework that guides nursing knowledge development and application (Koltoff, 2022). According to these authors, the absence of theory in higher education, and consequently in practice, is detrimental to a discipline. They propose the practical integration of knowledge, using the term *evidence-informed practice* as an alternative to *evidence-based practice*, emphasizing contextual and flexible integration of evidence. This approach considers scientific evidence as one among several sources of knowledge, including cultural values, local context, and practical knowledge.

Chinn et al. (2022) understand knowledge as a whole, which requires nurses to consider all standards of nursing knowledge as crucial for addressing patients’ particular needs and promoting patient-centred care. This model outlines five patterns (ways) of knowledge needed in nursing: (1) *Emancipatory knowledge* (social justice), (2) *ethical knowing* (the nature of right and wrong), (3) *personal knowing* (awareness of self and others), (4) *aesthetic knowing* (unique meaning and intent), and (5) *empiric knowing* (how things work and the nature of how nurses can know through experiences) (Chinn et al., 2022).

## Methods

This discussion paper presents a theoretical synthesis that bridges theory and practice in PIVC management in EDs. Tanner's CJM was critically appraised using Chinn and Kramer's evaluative criteria and integrated with their multidimensional Ways of Knowing to construct a comprehensive framework. The conceptual analysis was undertaken in October 2024 through iterative consensus discussions among the authors. A structured, theory-driven approach was adopted to conceptually evaluate the integration of Tanner's CJM using Chinn and Kramer's criteria and to explore the integration of these two complementary frameworks with Kramer's Ways of Knowing for PIVC decision-making and patient outcomes improvement in the ED. The application of these criteria transformed the appraisal from a subjective commentary into a systematic and replicable analysis. This structured approach ensures that the evaluation of Tanner's CJM is comprehensive and directly aligned with established standards for nursing theory, thereby strengthening the scholarly foundation of the subsequent integration.

These criteria—purpose, clarity, simplicity, generality, accessibility, and significance—were applied sequentially to assess the applicability and robustness of the CJM for guiding practice in the highly dynamic ED setting.

*Purpose* was examined by assessing the alignment of the CJM with contemporary ED nursing priorities; *clarity* by analysing the coherence and precision of its central concepts; and simplicity by evaluating its feasibility within fast-paced workflows. *Generality* and *accessibility* were assessed in terms of the model's adaptability across clinical contexts and the extent to which empirical indicators could be derived to support practice. The *significance* was evaluated based on the model's potential to enhance decision-making for PIVC management. Synthesis of these structured assessments enabled a coherent appraisal of Tanner's model, highlighting its strengths, limitations, and practical relevance to emergency care.

In parallel, it was employed an integrative strategy to explore how the CJM interacts with Chinn and Kramer's multidimensional Ways of Knowing. This involved mapping the abstract dimensions of empirical, ethical, personal, and aesthetic knowing onto practical decision-making steps in PIVC care. By examining how each knowledge pattern informs noticing, interpreting, responding, and reflecting, this integrative analysis demonstrated how theoretical constructs could be translated into actionable strategies tailored to the complexities of PIVC decision-making in the ED.

This dual-method approach—systematic appraisal followed by integrative mapping—ensures that the proposed framework is both theoretically sound and practically operational. The consensus-building process among the authors, involving iterative discussion and revision of the mappings presented in Table 2, served to challenge assumptions and refine the application of theory to the nuanced reality of ED-based PIVC care.

### Ethical Considerations

This paper is a theoretical discussion and does not involve the collection of primary data from human or animal participants. Therefore, ethical approval was not sought for this work. All information presented is derived from publicly available sources, cited appropriately to acknowledge original authors. The discussion aims to promote respectful, inclusive, and evidence-based dialogue, avoiding language or assumptions that could cause harm or perpetuate bias. The author(s) declare no potential conflicts of interest with respect to the research, authorship, and/or publication of this article.

## Results

### How Tanner's CJM Meets Chinn and Kramer's Specific Theoretical Evaluation Standards

To address the first objective of this paper, the following critical appraisal demonstrates how Tanner's CJM meets Chinn and Kramer's specific theoretical evaluation standards: Purpose, clarity and simplicity, generality, accessibility, and significance.

#### Purpose

Beginning with *purpose*, Tanner's CJM addresses the complexity of clinical decision-making in nursing practice by providing a structured approach to understanding and improving the cognitive processes involved in clinical judgment, thereby furthering the conceptual understanding of decision-making in nursing care. The model effectively informs nursing education and clinical practice by outlining the different stages and influencing factors in clinical judgment. Its theoretical rigour and practical applicability underscore its importance as a fundamental tool for enhancing critical thinking, reflective practice, and evidence-based decision-making within diverse healthcare settings (Tanner, 2006; Tanner et al., 2022).

#### Clarity and Simplicity

Regarding *clarity and simplicity*, the CJM is grounded in clear and simple core concepts of clinical judgment. The CJM is clearly articulated, outlining four specific phases—noticing, interpreting, responding (Tanner, 2006; Tanner et al., 2022)—making it accessible and implementable for nurses at various levels of practice. While the individual components are clear (Manetti, 2019), the dynamic interrelations and contextual nuances may not always be explicitly detailed (Dehghani, 2024; Nunes et al., 2020; van Graan et al., 2016). This can create ambiguity when applying the model to clinical contexts.

#### Generality

*Generality* is evident by the CJM's broad applicability across nursing fields and settings (Tanner, 2006; Tanner et al., 2022). This versatility supports its application in various nursing contexts, although some details may require local adjustment (Nevada State University, 2024).

#### Accessibility

*Accessibility* is a further aspect of this analysis. Tanner's model is accessible in terms of language and conceptual level, aimed at practising nurses, educators, and students. It is shared through nursing literature and education, making it widely available and understandable to its intended audience (Jessee et al., 2023; Lasater, 2007; Tanner, 2006).

#### Significance

The CJM is crucial in enhancing clinical judgment skills, promoting evidence-informed practice, and improving patient outcomes in complex healthcare environments (e.g., ED).

The theory addresses the complexity of clinical decision-making in nursing practice by providing a structured approach for understanding and improving the cognitive processes involved in clinical judgment (Tanner, 2006; Tanner et al., 2022). It articulates how nurses perceive, interpret, respond to, and reflect on patient information within complex clinical contexts. This approach is conducive to nursing education and practice, providing a clear framework for developing and assessing clinical judgment skills. This has a direct impact on patient outcomes and professional development.

The critical appraisal using Chinn and Kramer's criteria confirms Tanner's CJM as a robust and relevant framework for the clinical context under consideration. As summarized in Table 1, the model meets the core criteria for a useful nursing theory, providing a validated structure upon which to build an integrated approach to PIVC decision-making.

**Table 1.** Tanner's Clinical Judgment Model Evaluation According to Chinn & Kramer's Criteria and its Application in PIVC Management.

Chinn & Kramer's criteria	CJM Evaluation	Example on PIVC Management in ED
*Purpose*	Provides a theoretical framework to explain and enhance clinical judgment in nursing practice and education.	The CJM guides nurses in systematically assessing the need for PIVC insertion in the ED, supporting timely decision-making that balances clinical urgency with infection prevention and patient safety.
*Clarity and Simplicity*	Clearly outlines four phases of clinical judgment, though the dynamic relationships between phases may require contextual interpretation.	During the evaluation of the patient, the four CJM phases enable the nurse to *notice* DIVA, *interpret* the patient's hemodynamic status, *respond* by selecting an appropriate catheter size and site, and *reflect* on the effectiveness of the intervention.
*Generality*	Applicable across diverse nursing specialties and clinical settings, with flexibility for adaptation.	The CJM can be applied to PIVC decisions across varied EDs, including trauma and medical emergencies, allowing adaptation to different patient acuity levels.
*Accessibility*	Use clear, accessible language and is widely available in nursing education and literature.	The model's straightforward structure supports ED nurses with different experience levels in making evidence-informed decisions regarding PIVC insertion, maintenance, and removal.
*Significance*	Supports the development of clinical judgment and evidence-informed practice, contributing to improved patient outcomes.	Applying the CJM to PIVC management in the ED promotes early complication recognition (e.g., infiltration or phlebitis), thereby reducing catheter-related adverse events and improving patient outcomes.

PIVC = Peripheral intravenous catheter; ED = emergency department; CJM = Clinical Judgment Model; DIVA = difficult intravenous access.

Adapted from: Chinn and Kramer(2023); Tanner (2006). Tanner et al. (2022).

**Table 2.** Life-Cycle Evidence for PIVC Management Paired with Tanner's Clinical Judgment Model and Chinn & Kramer's Ways of Knowing.

Evidence-based stage of PIVC management	Tanner's Clinical Judgment Model	Chinn & Kramer's Ways of Knowing	Key decision considerations in the Emergency Department
Clinical assessment & justification	Noticing	Empirical & Ethical	- Verify a clear clinical indication for the PIVC - Weigh non-IV alternatives - Document the rationale for PIVC insertion or deferral
Pre-insertion planning	Noticing and Interpreting	Empirical & Aesthetic & Personal	- Assess the difficulty of venous access - Select an anatomically and therapeutically appropriate vein–catheter combination - Integrate the patient's prior cannulation history
Skin antisepsis & cannulation technique	Responding	Empirical & Ethical	- Perform evidence-based skin preparation - Choose standard or image-guided insertion according to access difficulty - Maintain sterility throughout cannulation
Securement & initial dressing	Responding and Reflecting (in-action)	Empirical & Aesthetic	- Apply an evidence-supported securement method - Choose the appropriate adhesive for the patient's skin so as not to cause damage - Label the dressing with essential insertion data
Maintenance	Responding and Reflecting (in-action)	Empirical & Ethical	- Adhere to a standardized flushing regimen - Conduct scheduled visual and tactile site assessments - Disinfect the access port before each use
Dwell time optimization & elective removal	Responding and Reflecting (on-action)	Empirical & Ethical	- Review PIVC necessity at predefined intervals - Act promptly on early signs of complication - Transition to a longer-term or alternative device when therapy duration warrants
Removal technique & post-removal surveillance	Responding and Reflecting (in-action)	Empirical & Ethical	- Follow a safe removal protocol - Ensure adequate hemostasis - Arrange post-removal site checks, with patient education on warning signs - Early removal of the PIVC, before medical discharge occurs
Quality improvement & disparity audit	Reflecting (system-level)	Emancipatory	- Monitor performance metrics stratified by patient and clinician characteristics - Reallocate resources to address inequities - Update training or policy based on audit findings - Structure simulation scenarios and guide students and nurses in developing clinical reasoning

PIVC = peripheral intravenous catheter; IV = intravenous.

Adapted from: Barrett and Carper (2022); Tanner (2006); Tanner et al. (2022).

**Figure 1.**
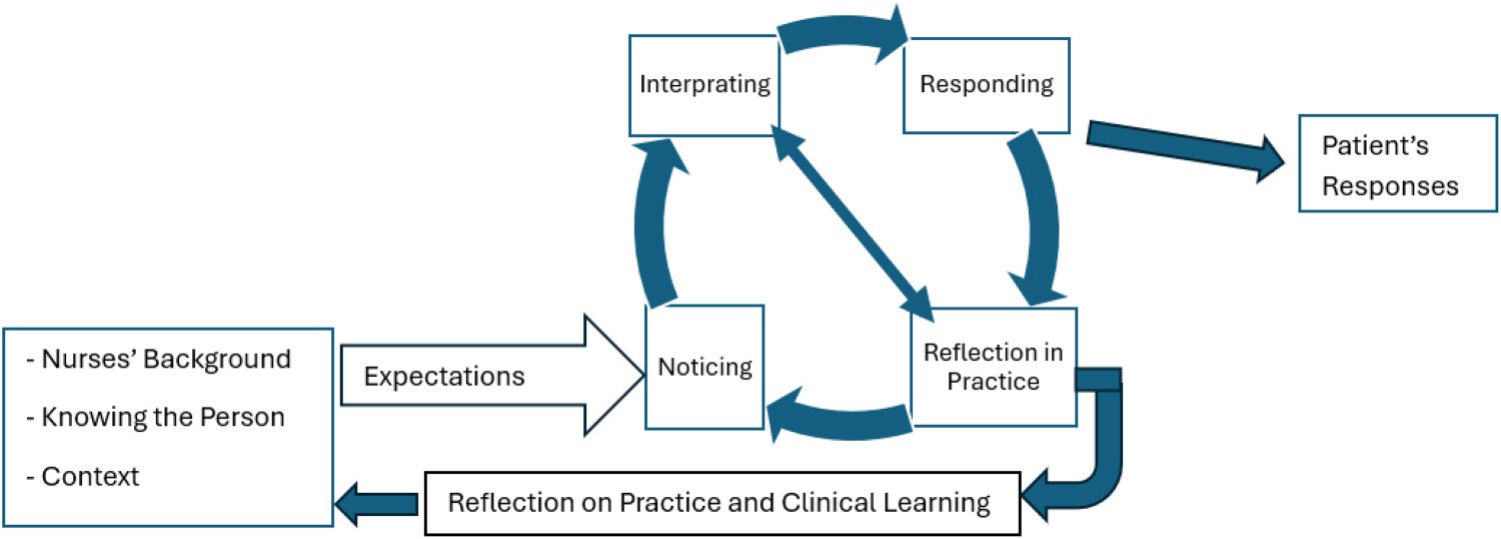
A research-based model of clinical judgment, adapted from Tanner's clinical judgment model.

The model possesses a clear and relevant purpose, is straightforward yet sufficiently comprehensive, adaptable across nursing contexts, accessible to its audiences, and essential in addressing key aspects of nursing practice (see Figure 1).

Having established that Tanner's CJM fulfils Chinn and Kramer's evaluative criteria, the following section discusses how the integration of these two frameworks can inform PIVC management in emergency care and enhance clinical decision-making in practice.

### Integrating Tanner's CJM and Chinn & Kramer's Ways of Knowing to Inform PIVC Management

Contemporary guidelines and high-level evidence demonstrate a consensus on a life-cycle approach to PIVC management from insertion to removal (Nickel et al., 2024; World Health Organization, 2024), transforming this procedure into a context-rich combination of actions. The integrated theory can inform practical strategies for improving decision-making around PIVCs and enhancing patient outcomes within the ED. Tanner's CJM provides the orientation to *when* each knowledge form is needed, while Ways of Knowing is a source of *what* knowledge forms must inform the specific moment.

The primary reflection that supports this work was based on triangulating various sources of evidence and different authors’ perspectives to structure this discussion: The four CJM phases (Tanner, 2006; Tanner et al., 2022), the Ways of Knowing (Chinn et al., 2022), and current guidelines (Gorski et al., 2021; Nickel et al., 2024). The integration of the two models yields a practical decision-support matrix, presented in Table 2. This matrix moves from abstract theory to actionable guidance by mapping the entire PIVC lifecycle against the cognitive phases of judgment and the specific types of knowledge required at each step. It serves as the core output of this theoretical synthesis, demonstrating how knowledge informs judgment to produce specific, evidence-informed actions.

## Discussion

When deciding to insert a PIVC in an ED setting, nurses engage in cognitive and reflective processes of clinical judgment throughout the *Noticing* phase (Chinn et al., 2022; Tanner, 2006; Tanner et al., 2022). During the clinical assessment prior to cannula selection, it must be confirmed that intravenous therapy is necessary and justified, and that no other delivery option is safer or more reliable (Gorski et al., 2021; Nickel et al., 2024). This process relies on empirical knowledge, which involves understanding the evidence-based benefits and risks of vascular access. *Ethical knowledge* requires prioritizing patient welfare by avoiding harm from unnecessary invasive devices (Chinn & Kramer, 2023; Chinn et al., 2022).

The pre-insertion planning stage comprises the initial observation and subsequent interpretation of relevant data, through *Noticing* and *Interpreting* phases. *Empirical knowledge* consists of an initial vascular assessment (e.g., vein diameter, palpability), but *personal knowledge* refines that assessment by including the patient's preference and history of venous access. *Aesthetic knowledge* in nursing is the intuitive, practice-based ability to perceive and interpret the clinical situation as a unified pattern rather than a collection of separate variables (Chinn & Kramer, 2023). During PIVC preinsertion planning, this form of knowing is essential because it operationalizes complex perceptual and relational data that empirical tools overlook (e.g., patient anxiety, aesthetic judgments about vein elasticity, venous trajectory, and anticipated limb movement); robust observational evidence links tacit expertise to significantly better insertion outcomes in ED settings (Carr et al., 2019). Combined, *noticing* and *interpreting* provide the foundation for integrating *empirical and personal knowledge* (mainly related to noticing) with *aesthetic knowledge* (more related to interpreting). This enables data assessment through pattern recognition, which guides device selection, such as choosing a short catheter versus a long guidewire or midline, or determining whether ultrasound guidance is necessary (Álvarez-Morales et al., 2024). At this stage, another contribution is introduced: The role of nursing intuition, which is linked to Chinn's *aesthetic knowledge* (Chinn et al., 2022; Chinn & Kramer, 2023) and was included by Tanner in the 2022-CJM (Tanner et al., 2022). This integration validates and demystifies the “art” of nursing. It positions intuition not as a mystical trait but as aesthetic knowing—a legitimate, experientially honed form of knowledge that operates in tandem with empirical evidence. By explicitly naming this within the framework, the researchers empower nurses to recognize, trust, and refine this critical aspect of their judgment, particularly in complex tasks such as assessing difficult venous access and preventing adverse patient outcomes (Manso et al., 2024; Ribeiro et al., 2024; Valenzuela, 2019). This interpretative and cognitive skill in acute care relies on pattern recognition developed through experience, emphasizing a thorough understanding of the patient, incorporating real-time reflection-in-action, and ongoing monitoring for early signs of deterioration (Tanner et al., 2022). Consistent with this, expertise is recognized as being influenced by previous experiences, educational background, and knowledge of the patients, all of which shape nursing surveillance (Benner, 2001; Benner et al., 2009; Tanner et al., 2022; Valenzuela, 2019).

This suggests that ED nurses should receive training in technical skills under the guidance of experienced nurses, which will impact the next phase—*Responding*.

In this phase, nurses perform deliberate, evidence-based actions grounded in clinical interpretation and analysis of patient needs (Dehghani, 2024; Tanner, 2006; Tanner et al., 2022), combining *empirical, ethical* and *aesthetic* knowledge (Chinn & Kramer, 2023). For example, choosing and applying the best antisepsis and insertion techniques is a clear, moment-to-moment response to assessing clinical risk (Clare & Rowley, 2021; Dehghani, 2024). Effective securement and the initial dressing are essential for maintaining PIVC site integrity, preventing microbial hazards, and reducing risks of dislodgement and local trauma (Corley et al., 2023; Zingg et al., 2023).

While empirical knowledge informs the choice of dressing, aesthetic knowledge ensures it is applied in a way that reduces pain, anxiety, distress, and is visually reassuring for the patient. For example, aesthetic knowledge is evident when a nurse arranges the dressing to suit a frail elderly person's delicate skin, aiming for comfort and dignity alongside physical security (Charters et al., 2024).

Therefore, ethical ways of knowing, combined with empirical understanding, ensure that every action, from handwashing to skin antisepsis or vein selection, protects the patient and maintains high standards of care, even under challenging circumstances (Chinn et al., 2022; Cuthbertson & Ashton, 2020; Plohal, 2021).

The maintenance stage of PIVC management encompasses flushing, monitoring and assessing, which directly involves *empirical* and *ethical* ways of knowing and aligns with *Responding* and *Reflecting* CJM phases. Predominantly within the same CJM domains, dwell-time optimization and elective replacement suggest *empirical* and *ethical* ways of understanding.

The guidelines emphasize routine flushing, using an appropriate pulsatile technique, and sufficient volume, vital for maintaining catheter patency, preventing occlusion, and reducing complications (Gorski et al., 2021; Keogh et al., 2020; Nickel et al., 2024). Specifically, ethical knowledge involves decisions about PIVC dwell time optimization and elective replacement, which require nurses to assess clinical necessity regularly, promptly remove the device when no longer needed, and involve the patient in decision-making where suitable (Ray-Barruel & Alexander, 2023).

Flushing, ongoing assessment, and timely intervention in response to complications (e.g., phlebitis, pain, or infiltration) exemplify clinical responsiveness. Nurses must decide, act, and document as situations evolve, a process guided by empirical evidence and ethical considerations. Regular assessment of dwell time and the option for elective replacement require nurses to consider the ongoing need and appropriateness of the device, reviewing previous interventions and patient outcomes to enhance future practice. This supports reflective practice and continuous quality improvement.

As shown in Table 2, although related to Tanner CJM's Reflecting phase, the two stages of PIVC management have a distinct focus. While maintenance involves reflecting *in action*, dwell time optimization and elective removal imply reflecting *on action*. This distinction is essential for nurses and other professionals to adapt in complex, dynamic environments and to continuously improve their practice through structured learning (Teixeira et al., 2025a). In the case of PIVC maintenance, when a nurse notices unexpected signs of a complication, they immediately re-evaluate and modify the intervention plan in real time to ensure patient safety. Evaluating dwell-time and the need for elective replacement, on the other hand, involve stepping back to examine actions, decisions, and outcomes to learn and improve future practice, implying reflection on action.

By raising awareness of the importance of reflection and distinguishing between *in action* and *on action*, Tanner's CJM (Tanner et al., 2022) fosters the opportunity for nurses to engage in deliberate training in reflective practice. The explicit distinction between *reflection-in-action* and *reflection-on-action* is a crucial contribution of this framework to PIVC management. It shifts quality improvement from a retrospective, audit-based activity to a dynamic and integrated component of daily practice. Encouraging *reflection-in-action* empowers nurses to make real-time adjustments, while structured *reflection-on-action* at the system level (e.g., during shift handovers or quality meetings) drives sustained practice change. Evidence findings also underscore the need for structured training programmes that integrate reflective practice principles to empower nurses to deliver safe, competent, and patient-centred PIVC care (García-Mayor et al., 2021; Hernon et al., 2023; Keleekai et al., 2016).

This suggests that ED nurses should receive training not only in technical skills but also in reflective and ethical reasoning.

Another part of the Responding phase, the removal technique and post-removal surveillance demand empirical and ethical knowledge to guarantee patient safety and optimal clinical outcomes. Nursing surveillance is a competence aimed at continuously acquiring, interpreting, and synthesizing the patient's clinical information, crucial for decision-making (International Council of Nurses, 2019; Meyer & Lavin, 2005; Tanner et al., 2022). Empirically, best practices requires using a standard aseptic technique during catheter removal to reduce infection risk and post-removal monitoring for site assessment of complications signs, such as bleeding, infection, or phlebitis, requiring timely intervention (Centers for Disease Control and Prevention, 2024; Zingg et al., 2023). Ethical knowledge underpins the necessity of removing the catheter as soon as it is no longer clinically needed or at the first sign of malfunction or adverse effects to prevent patient harm (Ray-Barruel et al., 2023). Furthermore, clear communication with the patient regarding the removal process and aftercare demonstrates respect for their autonomy (Registered Nurses’ Association of Ontario, 2019). Documenting the removal procedure is essential for accountability and continuity of care (Bahl et al., 2024).

Quality improvement and disparity audits are system-level Reflection phases that should include emancipatory and personal ways of knowing (Chinn & Kramer, 2011). Emancipatory knowing focuses on awareness and critical reflection of social, cultural, and political conditions that perpetuate inequities, to foster transformative action (praxis) toward social justice and equity in healthcare.

This stage is illustrated by nurses actively involving patients in decisions about their PIVC care, educating them about the purpose, risks, and signs of complications, and empowering them to voice concerns and participate in treatment decisions. This addresses power imbalances inherent in healthcare and fosters patient autonomy (Ray-Barruel et al., 2025). The same is noted when recognizing disparities in care quality or access to PIVC management resources among marginalized populations. Nurses also advocate for equitable resource allocation, standardized protocols, and culturally sensitive care to improve outcomes and reduce harm (Blanco-Mavillard et al., 2022; Hu et al., 2025). Finally, nurses critically examine organizational policies and practices around PIVC management, challenging outdated or harmful routines and promoting education, communication, and collaboration that align with social justice principles (Prasad et al., 2022; Ray-Barruel et al., 2025). Altogether, this leads to improved patient outcomes, including patients’ perceptions of the care provided by nurses, as captured through patient-reported outcome measures and patient-reported experience measures (Benson, 2023).

This paper demonstrates that in the absence of applied clinical judgment, guidelines and protocols become static checklists. With CJM, they become a dynamic, reproducible decision pathway capable of maintaining guideline fidelity amid the speed and complexity of emergency care (Tanner, 2006; Tanner et al., 2022). Chinn and Kramer's Ways of Knowing are equally vital, as they supply the epistemic energy that propels each cycle of CJM (Chinn et al., 2022; Chinn & Kramer, 2023).

Critical reflection on theory can assist nurses in understanding how this can be a valuable component of evidence-informed practice (Koltoff, 2022). The application of clinical judgment theory to PIVC management, demonstrated above, confirms that decision-making skills improve patient safety by supporting more appropriate, evidence-informed, and patient-centred decisions regarding PIVC insertion, maintenance, and removal. Combining these two Models allows us to directly address the two fundamental aims of emergency care: Technical proficiency and compassionate, equitable patient outcomes. When applied with ongoing feedback, this integrated framework can transform a traditionally routine procedure into a consistently safe, patient-centred practice.

Contemporary guidelines view PIVC management as a continuum beginning with clinical justification and ending with post-removal surveillance and quality audit. Respected guidelines (Gorski et al., 2021; Nickel et al., 2024), and high-level systematic evidence on infection control (Costa et al., 2026; Teixeira et al., 2025b; Zingg et al., 2023), all insist that insertion technique alone is insufficient. Instead, they locate safe PIVC use within a sequence of interlocking decisions on securement, flushing, dwell-time optimization, and timely removal, each explicitly tied to measurable outcomes and organizational accountability. This consensus underpins the decision to embed eight sequential stages inside an ED workflow, thereby operationalizing best practice from indication to audit.

### Strengths and Limitations

The analytical strength of this paper lies in its dual mapping of those eight stages of PIVC management into Tanner's CJM and Chinn & Kramer's multidimensional Ways of Knowing. Employing the CJM's Noticing, Interpreting, Responding, and Reflecting (Tanner, 2006; Tanner et al., 2022) and pairing each PIVC phase with empirical, ethical, aesthetic, personal, or emancipatory knowledge (Chinn & Kramer, 2023; Chinn et al., 2022) has clearly shown which forms of evidence should dominate a given clinical moment. For example, empirical data drive flushing regimens (Keogh et al., 2020), while aesthetic knowledge and intuition sharpen vein selection in difficult access patients (Carr et al., 2019). Ethical reasoning intersects every phase, whether in avoiding unnecessary *just-in-case* cannulas that harm one in four recipients (Marsh et al., 2024; Ray-Barruel & Alexander, 2023) or in de-escalating to safer modalities once therapy goals change (Ray-Barruel & Alexander, 2023). This integrative scaffold underscores that robust PIVC-related practice is technical, cognitive, relational, and moral. It implies a management process extending beyond *simple practice*.

Reflexively, this paper also recognizes praxis-oriented gaps that persist despite strong evidence: The underuse of reflective practice curricula (García-Mayor et al., 2021), inequities revealed through disparity audits (Blanco-Mavillard et al., 2022; Hu et al., 2025), and the contested status of nursing intuition in acute care (Ribeiro et al., 2024; Valenzuela, 2019). By foregrounding emancipatory knowing, nurses are invited to interrogate structural obstacles, like staffing ratios, supply chains and cultural safety, that modulate PIVC outcomes beyond the bedside. Such a critical stance aligns with Schön (1987, 2017) call for reflection-in-action and on-action, offering a pathway for continual improvement at individual and system levels. Overall, this discussion paper has woven disparate evidence strands into a coherent, justice-oriented roadmap that can scaffold clinical judgment, guide education, and inform policy in the fast-paced environment of the ED.

The primary contribution of this paper is therefore conceptual and heuristic. It provides a novel lens through which educators, clinicians, and researchers can conceptualize PIVC management. The proposed framework generates specific, testable hypotheses—for instance, that educational interventions explicitly teaching the integration of aesthetic and empirical knowing will improve first-attempt insertion success, or that audit tools structured around the phases of CJM will more effectively identify system-level barriers than checklist-based audits alone.

While this work provides a conceptual contribution, several limitations remain, highlighting the need for further research. Questions for future consideration include: Is there adequate access to evidence-based information in the clinical area? Are teaching methods of PIVC management appropriate? Do nurses underestimate the importance and risks of PIVCs? Could a greater focus on nurses’ decision-making skills improve PIVC care and patient outcomes? Future studies should apply Tanner's CJM to analyze the role of nurses’ experience in decision-making and clinical reasoning. Minor areas need further clarification or contextual adjustment, namely, its effectiveness in education, more substantial evidence regarding the role of experience, and its application to clinical reasoning in emergent situations (Tanner et al., 2022). They must involve reflection about decision-making in this area, with strategies adapted to the specific environment of the ED (Teixeira et al., 2025a). Better understanding of patients’ perceptions about the PIVC care provided is also needed.

### Implications for Practice

This paper emphasizes the importance of reflecting on compliance with theoretical knowledge and published guidelines to ensure quality care in PIVC management. It promotes critical reflection on Tanner's CJM, proposing how nurses can enhance PIVC management in the ED by integrating theory with practice to improve patient outcomes. The Chinn and Kramer Knowledge Model is applied to analyze Tanner's Theoretical Model, offering a framework for critical reflection on theory, which can guide nurses in refining their clinical decision-making process. When conflicts arise between empirical guidelines and aesthetic or personal knowing, nurses must engage in reflective clinical judgment to integrate scientific evidence with experiential knowledge, contextual factors, and the patient's specific clinical situation, thereby ensuring safe and well-justified clinical decisions. In the ED, this integrative approach Model is essential for adapting PIVC management to complex, time-sensitive situations. The alignment of CJM Model with Chinn and Kramer's Knowledge Model provides a robust bridge between theory and practice, supporting both educational and organizational reflection developed collaboratively within the clinical context. Strategies such as the *Journal Club* exemplify this integration by fostering critical appraisal of evidence, strengthening clinical reasoning, and facilitating the context-sensitive application of evidence-informed practice. Collectively, this approach enables the establishment of realistic clinical goals and directly impacts the quality and safety of emergency nursing care.

This paper could serve as a foundation for future research to evaluate the effectiveness of these models in education, using problem-based learning (Yew & Goh, 2016), Journal Club (Eusuf & Shelton, 2022) and debriefing (Reierson et al., 2017), as methodologies to improve *reflection in* and *on action* in this area. It could also be essential to encourage system-wide reflection about the role of experience and clinical reasoning in specific emergent situations and equitable resource allocation.

Ultimately, this integrated framework advocates for a paradigm shift: From viewing PIVC care as a series of discrete technical tasks to understanding it as a continuum of sophisticated clinical judgments. It argues for investing in nurse's cognitive and reflective skills with the same rigour applied to technical skills. This shift is essential for closing the persistent theory-practice gap and achieving reliable, high-quality, and patient-centred vascular access outcomes in the demanding emergency care environment.

## Conclusion

As nurses are the primary clinical workforce responsible for managing PIVC globally, understanding the processes underlying nurses’ clinical judgment is imperative. Incorporating Tanner's CJM and Chinn and Kramer's Ways of Knowing into PIVC management provides a useful scaffold for developing reflective clinical judgment skills. Current evidence supports extending this model to educational settings to evaluate its effectiveness in enhancing clinical training. This could be achieved using debriefing and problem-based learning methodologies to improve reflection in and on action.
